# A horizontally gene transferred copper resistance locus confers hyper‐resistance to antibacterial copper toxicity and enables survival of community acquired methicillin resistant *Staphylococcus aureus* USA300 in macrophages

**DOI:** 10.1111/1462-2920.14088

**Published:** 2018-03-26

**Authors:** Joanne Purves, Jamie Thomas, Gustavo P. Riboldi, Marta Zapotoczna, Emma Tarrant, Peter W. Andrew, Alejandra Londoño, Paul J. Planet, Joan A. Geoghegan, Kevin J. Waldron, Julie A. Morrissey

**Affiliations:** ^1^ Department of Genetics University of Leicester, University Road Leicester LE1 7RH UK; ^2^ Department of Infection, Immunity and Inflammation University of Leicester, University Road Leicester LE1 9HN UK; ^3^ Institute for Cell & Molecular Biosciences, Faculty of Medical Sciences Newcastle University, Framlington Place Newcastle upon Tyne NE2 4HH UK; ^4^ Department of Microbiology Moyne Institute of Preventive Medicine, School of Genetics and Microbiology, Trinity College Dublin Ireland; ^5^ Department of Pediatrics Columbia University New York, NY USA; ^6^ Department of Pediatrics, Perelman School of Medicine University of Pennsylvania Philadelphia PA USA

## Abstract

Excess copper is highly toxic and forms part of the host innate immune system's antibacterial arsenal, accumulating at sites of infection and acting within macrophages to kill engulfed pathogens. We show for the first time that a novel, horizontally gene transferred copper resistance locus (*copXL*), uniquely associated with the SCC*mec* elements of the highly virulent, epidemic, community acquired methicillin resistant *Staphylococcus aureus* (CA‐MRSA) USA300, confers copper hyper‐resistance. These genes are additional to existing core genome copper resistance mechanisms, and are not found in typical *S. aureus* lineages, but are increasingly identified in emerging pathogenic isolates. Our data show that CopX, a putative P_1B‐3_‐ATPase efflux transporter, and CopL, a novel lipoprotein, confer copper hyper‐resistance compared to typical *S. aureus* strains. The *copXL* genes form an operon that is tightly repressed in low copper environments by the copper regulator CsoR. Significantly, CopX and CopL are important for *S. aureus* USA300 intracellular survival within macrophages. Therefore, the emergence of new *S. aureus* clones with the *copXL* locus has significant implications for public health because these genes confer increased resistance to antibacterial copper toxicity, enhancing bacterial fitness by altering *S. aureus* interaction with innate immunity.

## Introduction

New methicillin resistant *Staphylococcus aureus* (MRSA) clones are emerging and circulating worldwide presenting a new threat to human health. The reasons for the emergence of these clones are currently unknown. *S. aureus* is an opportunistic pathogen responsible for a range of minor and life‐threatening diseases (Sullivan *et al*., 1984; Tong *et al*., 2015) and is considered by the World Health Organisation to be one of the major multidrug resistant bacterial health threats globally (WHO, 2017). Hospital‐associated (HA) MRSA are a leading cause of nosocomial infections worldwide, predominantly infecting patients with reduced immune function. However, the MRSA problem has been exacerbated by the emergence and spread of community‐acquired (CA) MRSA, which can cause infections in healthy humans with no previous exposure to healthcare situations (Otto, 2013). CA‐MRSA mainly causes severe skin and soft tissue infections (SSTI; Johnson *et al*., 2007) but can also cause invasive life‐threatening infections, for example necrotizing pneumonia (Francis *et al*., 2005).

The CA‐MRSA clone USA300 is epidemic in the United States (Mediavilla *et al*., 2012). The success of USA300 has been attributed in part to increased expression of virulence genes and the acquisition of several mobile genetic elements (MGE) encoding genes that enhance USA300 colonization, virulence and environmental survival (Diep *et al*., 2006). *S. aureus* USA300, and other CA‐MRSA isolates, also show increased resistance to innate immunity and reduced clearance from healthy airways compared to other *S. aureus* clinical isolates (Yajjala *et al*., 2016). This has been attributed to elevated resistance to alveolar macrophage‐mediated intracellular killing, although the exact mechanisms involved have not been established (Yajjala *et al*., 2016).

The North American USA300 clone (USA300‐NAE) has spread globally (Glaser *et al*., 2016), and a Latin American variant (USA300‐SAE) is epidemic in South America (Nimmo, 2012). Interestingly, phylogenetic analysis determined that USA300‐NAE and USA300‐SAE evolved from a common ancestor, with individual parallel epidemics emerging simultaneously (Planet *et al*., 2015). USA300‐SAE cases are now being reported in North America and Europe (Dach *et al*., 2016; Planet *et al*., 2016) suggesting that this clone is also spreading globally.

**Figure 1:**
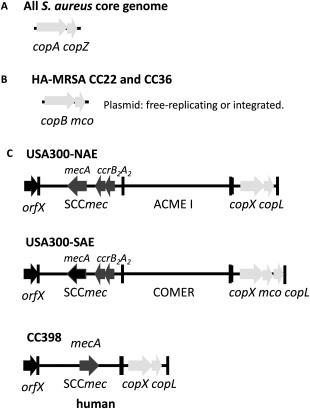
The structure of *S. aureus* copper resistance operons. A. The archetypal *S. aureus copAZ* operon encoding *copA*, a copper‐translocating P‐type ATPase and *copZ*, a copper metallochaperone found in the core genome of all *S. aureus* strains. B. The *S. aureus copBmco* operon encoding a putative copper‐translocating P‐type ATPase and a multicopper oxidase respectively carried on a plasmid that can be either chromosomally integrated or free replicating. C. The location of the *copX* and *copL* genes, encoding a putative copper‐translocating P‐type ATPase and a lipoprotein of unknown function respectively, in different *S. aureus* lineages.

Both USA300 variants cause the same range of diseases and have become the predominant epidemic clones. Acquisition by horizontal gene transfer (HGT) of the arginine catabolic mobile element (ACME; Thurlow *et al*., 2013) was suggested to be key to the success of USA300‐NAE. However, USA300‐SAE isolates do not encode ACME but instead carry a novel MGE encoding putative copper and mercury resistance (COMER) determinants (Fig. 1; Planet *et al*., 2015). Across the ACME and COMER elements of the USA300‐NAE and USA300‐SAE clades, there are only two genes that are conserved, SAUSA300_0078 and SAUSA300_0079, encoding a putative efflux transporter and a putative lipoprotein respectively (Fig. 1; Planet *et al*., 2015). Consequently we hypothesized that this novel locus may play a role in copper resistance, and potentially the enhanced fitness and success of the USA300 lineage by increasing survival against antimicrobial copper.

Copper is an essential nutrient as a cofactor for some essential enzymes, but excess copper is highly toxic to bacteria (Djoko *et al*., 2015). Copper accumulates at sites of infection, where it plays a major role in innate immunity, with the infected host using copper to kill invading microorganisms (Beveridge *et al*., 1985; Fones and Preston, 2013; Chaturvedi and Henderson, 2014; Fu *et al*., 2014; Johnson *et al*., 2015; Hyre *et al*., 2017). Importantly macrophages, which are critical to innate immunity, protecting the skin and respiratory tract (Malissen *et al*., 2014; Kopf *et al*., 2015), use copper as an antibacterial mechanism by actively importing it into the phagosome (White *et al*., 2009; Johnson *et al*., 2015). Consequently pathogens have mechanisms to counteract copper toxicity, mainly by limiting the copper concentration in their cytoplasm through efflux or sequestration, which are important for virulence in a diverse range of bacterial pathogens in a variety of infection models (Schwan *et al*., 2005; Ward *et al*., 2010; Shafeeq *et al*., 2011; Johnson *et al*., 2015; Hyre *et al*., 2017; Ladomersky *et al*., 2017).

All *S. aureus* possess a conserved operon encoding a P_1B‐1_‐type ATPase copper efflux transporter (*copA*) and a copper chaperone protein *(copZ)* (Sitthisak *et al*., 2007; Fig. 1A) encoded as part of the core genome. The role of *S. aureus* CopA in virulence is unknown. Some HA‐MRSA (CC36 and CC22 lineages) also possess an additional copper exporting ATPase belonging to the P_1B‐3_ subtype, designated *copB*, encoded on a plasmid that is either freely‐replicating or integrated into the genome (Fig. 1B; Baker *et al*., 2011). The *copXL* genes encoded in both the ACME and COMER elements are predicted to encode a novel copper resistance locus encoding a putative P_1B‐3_‐type ATPase (SAUSA300_0078; herein designated *copX*) and a putative lipoprotein (SAUSA_0079, herein designated *copL)*.

We have studied the function of this putative copper resistance locus (*copXL*) and found that it confers copper hyper‐resistance and promotes survival of USA300 in macrophages. *S. aureus* isolates carrying the *copXL* locus showed significantly increased copper resistance relative to strains with only the conserved *copAZ* operon. Cells of isogenic mutants of *S. aureus* lacking *copX* showed decreased resistance to copper, and cells lacking either *copX* or *copL* showed increased copper accumulation, suggesting that both genes play a role in copper efflux, with CopX likely being a copper‐specific efflux transporter. Transcriptional analysis demonstrated that expression of the *copXL* operon is induced by copper, and regulated by the copper sensor CsoR, which specifically binds to the promoters of both *copAZ* and *copXL*. Importantly, the *copXL* locus also plays a key role in survival against killing by macrophages, demonstrating the importance of *copXL* for increased resistance of *S. aureus* USA300 to innate immune defences.

## Results

### *S. aureus* USA300 encodes a novel copper resistance locus

The *copXL* locus shares 99% DNA sequence identity with a locus from coagulase negative *Staphylococcus* species, suggesting it has recently been acquired by *S. aureus* USA300 through HGT. In USA300‐SAE, the *copXL* genes are found in COMER, associated with the staphylococcal cassette chromosome *mec* (SCC*mec*) element and a multi‐copper oxidase (*mco*), whereas in USA300‐NAE the *copXL* genes are directly adjacent to the SCC*mec*/ACME element (Fig. 1C). Genome searches confirmed that these genes are not found in typical *S. aureus* lineages such as methicillin sensitive *S. aureus* (MSSA). However, there is increasing evidence that some other clinical isolates have recently independently acquired *copXL* genes with 99% DNA sequence identity to the USA300 *copXL* genes, e.g., human and livestock‐associated CC398 (Gómez‐Sanz *et al*., 2014; Ward *et al*., 2014; Fig. 1C), Western Australia MRSA‐59 (Monecke *et al*., 2015), and Irish healthcare associated ST779 (Kinnevey *et al*., 2013), suggesting that there is currently a strong selection for acquisition of these genes.

**Figure 2:**
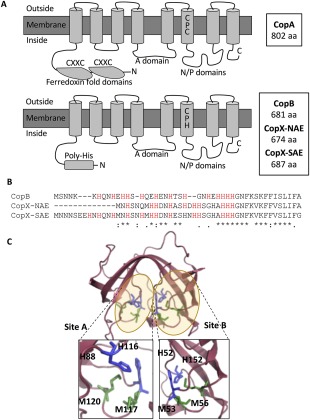
Schematic of *S. aureus* CopA and CopX P_1B_‐type ATPase transporters showing conserved eight transmembrane domains, phosphatase (A) domains and ATP binding/phosphorylation (N/P) domain. CopA has 2 ferredoxin domains and CxxC copper binding motifs, whereas CopX has a novel amino‐terminal poly‐His sequence. B. Alignment of CopB and CopX amino‐terminal poly‐His sequences. C. Predicted structural model of CopL. Modelling of the CopL structure built with Swiss‐Model based on 4MDW shows that conserved His and Met residues form two spatial clusters which we hypothesize form copper binding sites within the ‘horseshoe’ structure of the tandem DUF1541 domains. Protein backbone structure is shown as a ribbon, and predicted ligands (Met = green, His = blue) of putative copper binding sites (highlighted yellow) are shown as sticks.

The CopX protein is a predicted P_1B‐3‐_type ATPase efflux transporter (Fig. 2A; Odermatt *et al*., 1993; Mana‐Capelli *et al*., 2003; Meloni *et al*., 2014). CopX shares 36% identity overall with the archetypal *S. aureus* CopA polypeptide (Sitthisak *et al*., 2007) but shares 83% overall identity with CopB, which is found only in the genomes of a subgroup of *S. aureus* lineages (Baker *et al*., 2011; Fig. 1B). All three proteins have the characteristic eight transmembrane domains including the key CPX motif, a conserved E1‐E2 ATPase domain and a carboxyl terminal haloacid dehalogenase‐like hydrolase domain. CopA has two amino terminal ferredoxin–like, heavy metal associated (HMA) domains and corresponding CxxC copper binding motifs, whereas both CopX and CopB lack these domains, instead possessing predicted intracellular amino‐terminal histidine‐rich metal binding domains common to the P_1B‐3_ subtype of this protein family (Fig. 2A; Odermatt *et al*., 1993; Mana‐Capelli *et al*., 2003; Meloni *et al*., 2014). Alignment of CopX and the other *S. aureus* copper pumps that excluded the N‐terminal domains showed similar levels of homology (37% identity to CopA, 86% identity to CopB), whereas an alignment of just the His‐rich N‐terminal domains of CopX and CopB showed just 59% identity (Fig. 2B).

The CopL polypeptide is predicted to be a surface‐exposed lipoprotein that contains two novel tandem domains of unknown function (DUF1541). Homologues of CopL are highly conserved in Staphylococci spp. and are also present in other Gram‐positive bacteria found in oceans, estuaries and soil but no CopL homologues have been functionally characterized, and thus the functions of these proteins and their domains are unknown. However, *in silico* analysis identified homology (51% identity) to an uncharacterized protein from *Bacillus subtilis* YdhK, whose structure has been determined by X‐ray crystallography (PDB ID 4MDW). Modelling (SWISS‐Model; Biasini *et al*., 2014) of the *S. aureus* CopL structure based on YdhK suggested that a number of conserved His and Met residues may form two spatial clusters that could form potential copper binding sites within the ‘horseshoe’ structure of the tandem DUF1541 domains (Fig. 2C).

**Figure 3:**
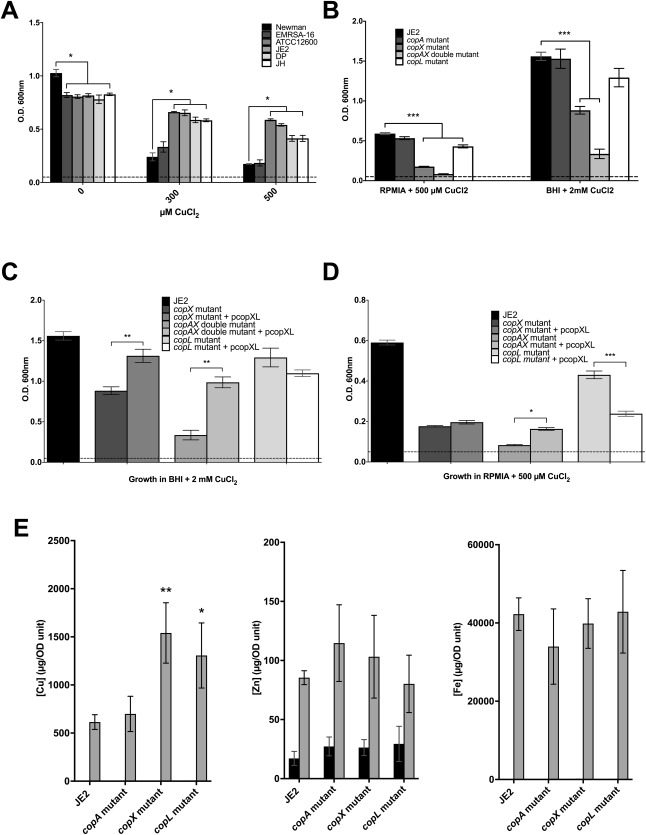
The effect of copper on the growth of *S. aureus* isolates encoding different putative copper resistance systems. Growth of (A) wild type *S. aureus* strains Newman (*copAZ*), EMRSA‐16 (*copAZ*; chromosomal encoded *copBmco*), ATCC12600 (*copAZ*; plasmid encoded *copBmco*), JE2 (*copAZ*; *copXL*), 078588D (DP; *copAZ*; *copXL*) and 072736J (JH; *copAZ*; *copXL*) cultured in RPMI‐A with 0, 300 and 500 µM CuCl_2_; (B) JE2 and *copA*, *copX, copL* and *copAX* mutants in both RPMIA with 500 µM and BHI with 2mM CuCl_2_; (C) the copper mutants carrying the pcopXL complementation plasmid in BHI with 2mM CuCl_2_ and (D) RPMI‐A with 500 µM CuCl_2_. In each case, optical densities at 600 nm were determined after 24 h of growth at 37°C for 24 h in 5% (v/v) CO_2_. Error bars represent ± 1 SEM of at least 3 independent biological repeats. Significance of JE2 growth compared to other strains was determined with a two‐way ANOVA test (* = *p* ≤ 0.05, ** = *p* ≤ 0.01, *** = *p* ≤ 0.0001). Dashed lines on graphs represent the starting optical density. (E) Whole cell metal content as determined by ICP‐MS analysis for (*left*) copper, (*centre*) zinc and (*right*) iron of JE2 and mutant cells of *S. aureus* cultured in RPMI‐A in the absence (black bars) or presence of an elevated but subinhibitory concentration (100 µM) of copper, zinc or iron respectively. Metal levels are shown as mean ± SEM from at least 3 independent biological repeats. (* = *p* ≤ 0.05, ** = *p* ≤ 0.01).

To test the hypothesis that *copXL* is a novel copper resistance locus, we measured the growth of several *S. aureus* strains of various genotypes (*copXL* ^+^ vs. *copXL* ^‐^) in elevated concentrations of CuCl_2_ in the defined minimal medium RPMI‐A. Our data show that *S. aureus* strains encoding a single chromosomal copy of the *copXL* locus, in addition to the core genome conserved *copAZ* operon, show copper hyper‐resistance compared to typical *S. aureus* strains (*p* ≤ 0.0001; Fig. 3A). The *copXL^+^* strains, MRSA USA300 JE2, the CC398 MRSA porcine isolate 078588D (DP; Ward *et al*., 2014) and the CC398 MRSA human isolate 072736J (JH; Ward *et al*., 2014) all showed a significant increase in growth in high copper compared to *S. aureus* Newman (*p* ≤ 0.0001; Fig. 3A). *S. aureus* Newman, which encodes only the core *copAZ* locus with no additional copper resistance elements, has copper resistance typical of the majority of *S. aureus* lineages (Baker *et al*., 2011).

EMRSA‐16 and ATCC12600 both encode the additional *copBmco* locus, EMRSA‐16 as a single copy on the chromosome, whereas ATCC12600 encodes the *copBmco* locus on a multicopy plasmid (Baker *et al*., 2011). The difference in gene copy number is reflected in copper resistance because ATCC12600, unlike EMRSA‐16, shows a significantly increased growth in 300–500 µM copper compared to Newman (*p* ≤ 0.0001; Fig. 3A). Interestingly, copper resistance of USA300 JE2, with its single chromosomal copy of the *copXL* locus, is comparable to ATCC12600, which possesses the *copBmco* locus on a multicopy plasmid. While it is possible that other factors may contribute to these phenotypes, these data suggest that the *copXL* locus may be a more effective copper resistance mechanism than a single copy of *copA* or *copBmco*.

It should be noted that all MRSA strains and the copper hyper‐resistant *S. aureus* ATCC12600 showed a growth defect in low copper conditions compared to the methicillin sensitive strain, *S. aureus* Newman (*p* ≤ 0.0001; Fig. 3A), although whether this fitness cost is due to the presence of these additional copper resistance loci or another, unrelated factor is unclear.

### The *copXL* locus confers hyper‐copper resistance

To investigate the role of *copXL* in copper resistance, *S. aureus* JE2 isogenic *copX, copL, copA* and *copXA* insertion mutants were constructed using *S. aureus* JE2 transposon library mutants, as described previously (Bose *et al*., 2013). All mutations were transduced to a clean JE2 background and all copper‐related phenotypes were maintained after transduction and were similar to the original transposon.

Our data show that mutation of the *copX* gene significantly increases *S. aureus* JE2 sensitivity to copper in the restricted medium RPMI‐A, and in the rich complex medium BHI (*p* ≤ 0.0001; Fig. 3B). Interestingly, the *copL* mutant showed a significant copper‐dependent growth defect in RPMI‐A (*p* ≤ 0.0001; Fig. 3B) but showed only a minimal growth defect in BHI (*p* = 0.06; Fig. 3B). Together these demonstrate that CopX is not dependent on CopL to function. Unexpectedly, the *S. aureus* USA300 JE2 *copA* mutant did not show significantly increased copper sensitivity in either media. The double *copAX* mutant, however, showed an increased sensitivity to copper compared to the *copX* mutant demonstrating that CopA does play a role in copper resistance in USA300, which is masked by CopX activity. None of the mutants showed a growth defect in the absence of added copper (Supporting Information Fig. S1).

To confirm that *copXL* confers copper hyper‐resistance in USA300, the *copX, copL* and *copAX* mutants were complemented by expression of a wild type copy of the *copXL* locus controlled by its native promoter encoded on pMK4 (pcopXL). Growth phenotype analyses showed significant complementation of the *copX* and *copAX* mutants in BHI, formally demonstrating that *copXL* does indeed confer copper hyper‐resistance in USA300 (*p* ≤ 0.01, Fig. 3C). However complementation of the *copL* mutant with pcopXL appeared to increase the sensitivity of the *copL* mutant to copper in BHI (Fig. 3C), and particularly RPMI‐A (Fig. 3D, *p* < 0.0001), suggesting that pcopXL can inhibit growth. This growth inhibition is copper‐dependent, because there are no differences in growth between any of the strains in BHI or RPMI‐A in the absence of additional copper (Supporting Information Fig. S1). Interestingly, the pcopXL plasmid also inhibited the growth of the *copX* and *copAX* mutants in the restrictive medium RPMI‐A because both complemented mutants only showed a small increase in growth in the presence of copper in RPMI‐A (*copAX p* ≤ 0.04, Fig. 3D), unlike in BHI.

### The *copX* and *copL* genes are important for copper efflux

To determine whether CopX functions as a copper efflux transporter, the whole cell metal content of wild type JE2 and the *copA* and *copX* mutant strains were compared by ICP‐MS analysis of extracts from acid‐digested cells after culture in RPMI‐A medium in the presence or absence of subinhibitory 100 µM CuCl_2_, Fe_2_(SO_4_)_3_ or ZnCl_2_ (Fig. 3E). As predicted the *copX* mutant contained significantly elevated copper content relative to the JE2 parent control, consistent with the hypothesis that *S. aureus* CopX is a copper efflux protein (*p* ≤ 0.04; Fig. 3E). The cellular iron or zinc content was unchanged in the *copX* mutant strain (Fig. 3E), which correlates with our inability to detect any phenotype of this mutant strain during growth analyses in the presence of inhibitory concentrations of zinc, nickel, manganese and iron (1–3 mM) (data not shown), suggesting that CopX is a copper‐specific resistance mechanism. Surprisingly, the *copA* mutant showed no change in cellular copper even though CopA is a known copper efflux protein, and has been shown to be essential for copper resistance for *S. aureus* strains that lack accessory copper resistance loci (Sitthisak *et al*., 2007; Fig. 3E). Thus, these data indicate that CopX is a highly effective efflux mechanism, and plays a major role in copper resistance in USA300.

ICP‐MS was also used to determine whether the novel CopL protein has a role in copper efflux. Unexpectedly, considering that CopL is a predicted surface‐bound lipoprotein, copper concentrations in the *copL* mutant cells were also elevated (*p* ≤ 0.05, Fig. 3E) despite the CopA and CopX efflux proteins both being functional in the *copL* mutant, showing that CopL likely functions in copper resistance and that CopX and CopL can act independently.

### The horizontally gene transferred *copXL* locus is regulated by copper and CsoR

Copper regulation of gene expression in *S. aureus* is mediated by the copper sensing transcriptional repressor CsoR (Baker *et al*., 2011). To determine whether the expression of *copXL* is regulated by copper and by CsoR in *S. aureus*, quantitative reverse transcriptase PCR (qRT‐PCR) was used to compare the transcription of the *copX* and *copA* genes in *S. aureus* JE2 and the JE2 *csoR::ΦΝΣ* transposon mutant strain, grown in RPMI‐A with and without subinhibitory 100 µM CuCl_2_ for 4 h. The JE2 *csoR* mutant strain showed no growth defect in elevated copper concentrations (data not shown), demonstrating that any impact on gene expression in the *csoR* mutant is due to a lack of CsoR repression. Analysis by qRT‐PCR using intergenic *copX* and *copL* primers, showed that the two genes are transcribed as a single transcript, and *copL* expression paralleled *copX* expression when tested independently (Supporting Information Fig. S2), confirming that the *copX* and *copL* genes form a bi‐cistronic operon.

**Figure 4:**
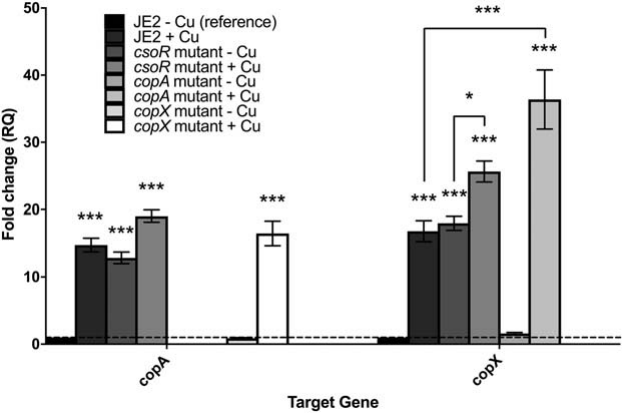
*CopXL* operon is repressed in low copper conditions. Transcription of *copA* and *copXL* was determined by qRT‐PCR in cells cultured in the presence or absence of subinhibitory CuCl_2_ (100 µM) during exponential growth in JE2, *copA*, *copX* and *csoR* mutants. Relative expression was calculated as RQ using the ΔΔCt method, which normalizes expression in each strain against an endogenous control gene (*gyrB*) and expresses the data relative to a reference strain (JE2 in the absence of Cu; RQ = 1 represented by the dashed line). Error bars represent ± 1 SEM of 3 biological repeats (each performed in technical triplicate). Significance of expression in each strain compared to the reference, or between strains of interest, was determined by 2‐way ANOVA with Dunnett's multiple comparison test (* = *p* ≤ 0.1, *** = *p* ≤ 0.0001).

The transcription of the *copXL* operon was regulated in a similar manner to the core genome *copA* gene. Transcription of *copX* was induced 17‐fold, and *copA* 15‐fold by copper (Fig. 4; *p* ≤ 0.0001), with both genes being repressed in low copper by CsoR, indicated by an 18‐fold and 13‐fold increase in expression in the *csoR* mutant in the absence of copper (Fig. 4; *p* ≤ 0.0001). Interestingly, both *copA* and *copXL* expression still showed some induction by copper in the *csoR* mutant, although only the *copXL* transcript showed a significant increase compared to low copper conditions (Fig. 4; *p* ≤ 0.01), suggesting that there is additional positive copper regulation in *S. aureus* USA300.

To investigate why the *copA* mutant strain showed negligible changes in copper efflux or growth, the transcription of the *copA* and *copX* genes in the reciprocal *copA* and *copX* mutants was assessed. Importantly, we detected a 40‐fold copper‐induced increase in *copXL* expression in the *copA* mutant (Fig. 4; *p* ≤ 0.0001) compared to a 15‐fold induction in the JE2 wild type strain. However, there was no reciprocal increase in *copA* expression in the *copX* mutant (Fig. 4) relative to JE2 in the presence of copper. Induction of *copXL* expression in the *copA* mutant presumably explains why the *copA* mutant showed no growth defect in high copper concentrations, because the increased expression of CopX could compensate for loss of CopA.

Transcription analysis showed that *copL* expression was found to be decreased in the *copX* insertion mutant, however, the gene is still expressed (Supporting Information Fig. S2). Additionally phenotypic analysis of a USA300 FPR3757 silent *copX* deletion mutation, which has a wild type level of *copL* transcription, had a similar phenotype to the *copX* insertion mutant, demonstrating that CopL is expressed at a sufficient level in the *copX* insertion mutant that its function is unaffected (Supporting Information Figs. S1 and S2).

To determine why the complemented strains showed growth inhibition in RPMI‐A, transcription of *copX* was also assessed in the complemented pcopXL *copX* insertion mutant compared to wild type JE2. Surprisingly *copX* expression was induced 37‐fold in low copper and 88‐fold in this strain in the presence of copper compared to wild type JE2 in the absence of copper (Supporting Information Fig. S2). This substantial increase in *copX* expression could potentially explain the observed inhibition of growth in in the complemented strains in high copper.

### CsoR directly binds to the *copXL* promoter to regulate copper‐dependent expression

**Figure 5:**
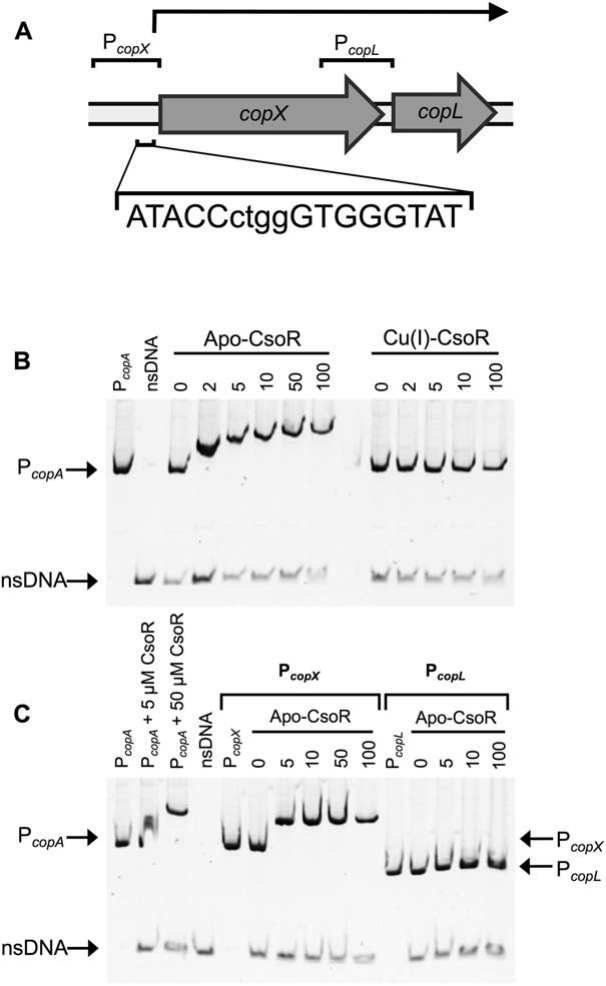
CsoR binds to the *copX* but not the *copL* promoter sequence *in vitro*. A. A schematic representing the position and sequence of the proposed CsoR binding site in the promoter region of *copX*. EMSA analysis was performed to test for binding of purified recombinant CsoR protein to the promoter sequences of the *copXL* locus. PCR products (∼200 bp) containing the sequences upstream of (B) *copA* (P_*copA*_), (C) *left*, *copX* (P_*copX*_), and *right*, *copL* (P_*copL*_), were mixed with a PCR product of non‐specific DNA sequence (nsDNA), and probed for binding of purified recombinant (A and B) apo‐ and (A only) Cu(I)‐CsoR.

Direct regulation of the *copX* and c*opL* putative promoters by binding of CsoR was tested by electrophoretic mobility shift analysis (EMSA) (Fig. 5). Putative CsoR binding sequences were identified in the promoters of *copA* (ATACCtataGGGGGTAC) and *copX* (ATACCctggGTGGGTAT), but there is no obvious binding motif in the 20 bp intergenic region between *copX* and *copL* (Fig. 5A). Recombinant CsoR was produced in *Escherichia coli*, purified to homogeneity by liquid chromatography, and used in anaerobic assays to characterize its binding to ∼200 bp DNA fragments containing the sequences upstream of *copA* (P_*copA*_), *copX* (P_*copX*_) or *copL* (P_*copL*_). As expected (Liu *et al*., 2007; Smaldone and Helmann, 2007), metal‐free CsoR showed strong binding to the PCR product encompassing the *copA* promoter, strongly retarding the migration of P*copA* in a dose‐dependent manner (Fig. 5B). In contrast, this DNA binding was absent after anaerobic incubation of CsoR with one equivalent of Cu(I) prior to addition of DNA (Fig. 5B). A PCR product of the sequence upstream of *copX* was also strongly retarded in its gel migration by apo‐CsoR (Fig. 5C), demonstrating specific binding of CsoR to the *copX* promoter. Conversely, the migration of a PCR product of the sequence upstream of *copL* (encompassing the 3’ end of the *copX* gene and the short intergenic region) showed no retardation (Fig. 5C), demonstrating no binding of CsoR to a putative *copL* promoter, confirming the qRT‐PCR data (Supporting Information Fig. S2) that the two genes are co‐transcribed. Therefore, expression of the *copXL* operon is repressed by direct binding of CsoR to the binding motif in the promoter upstream of *copX* under low copper conditions, and becomes de‐repressed under elevated copper conditions.

### CopX plays a major role in USA300 macrophage survival

**Figure 6:**
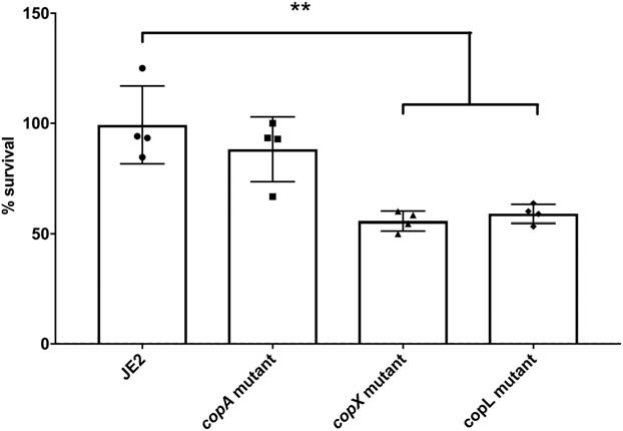
Survival of *S. aureus* from intracellular killing by macrophages. Mouse RAW264.7 macrophages were seeded at 2 × 10^6^ per ml in DMEM into 24‐well plates and activated with mouse IFN‐γ and Cu_2_SO_4_ for 18 h. Bacteria were added at a multiplicity of infection (MOI) of 10 in DMEM and co‐incubated with monolayers for 30 min. The monolayers were subsequently washed and extracellular bacteria were killed by treatment with gentamycin (200 µg ml^−1^) and lysostaphin (100 µg ml^−1^) for 30 min. To quantify intracellular bacteria, monolayers were washed and lysed with ice‐cold water at time point 0 (T0) and after 3 h (T3). CFUs were counted to determine numbers of viable bacteria. Bars represent the mean percentage survival ± SD for at least three independent experiments. (** = *p* ≤ 0.001).

*S. aureus* can survive phagocytic killing by macrophages (Kubica *et al*., 2008) but a role for copper resistance in the intracellular survival of *S. aureus* has not been described. A mechanism of copper‐enhanced bactericidal activity in macrophages was previously reported by White and colleagues (2009) whereby IFNγ‐activated upregulation of the copper importer, CTR1, and intracellular trafficking of the ATP7A transporter leads to enhanced killing of copper sensitive *E. coli* mutants by macrophages. To investigate the relevance of *copXL*‐ mediated copper hyper‐resistance to the ability of *S. aureus* to survive in macrophages, IFNγ‐activated macrophage survival experiments were performed. The levels of survival (CFU ml^−1^) of intracellular USA300 JE2 and the mutant strains lacking *copA*, *copX* or *copL* were compared 3 h after phagocytosis (Fig. 6). There was no difference in uptake of the mutants by macrophages as assessed by CFU at T0 (Supporting Information Fig. S3). Intracellular numbers of the wild type remained high at this time point contrasting with all of the copper sensitive mutants, which showed reduced survival. Both the *copX* and *copL* mutants showed a statistically significant decrease in survival (Fig. 6; *p* ≤ 0.001), suggesting an important role of the CopX copper exporter and CopL lipoprotein in overcoming copper toxicity following macrophage phagocytosis (Fig. 6).

## Discussion

In this study we demonstrate that the horizontally transferred *copX* and *copL* genes, confer copper hyper‐resistance, are repressed in low copper concentrations via the core genome copper‐dependent regulator CsoR, and play a key role in *S. aureus* survival in macrophages. The *copXL* genes are the only two genes common to the ACME and COMER elements associated with the SCC*mec* of both epidemic USA300‐SAE and USA300‐NAE strains of *S. aureus*. Together, these data suggest that the recent evolution and success of these epidemic MRSAs could be due to possession of these additional copper resistance genes, enhancing bacterial fitness both in the environment and within the host, and thus increasing the success of the USA300 lineage by increasing survival against the antimicrobial toxicity of copper.

Our data suggest that CopX is a likely copper efflux transporter of the P_1B‐3_ type (Odermatt *et al*., 1993; Mana‐Capelli *et al*., 2003; Meloni *et al*., 2014) responsible for *S. aureus* copper hyper‐resistance as confirmed by growth in both rich (BHI) and restrictive (RPMI‐A) growth conditions, and ICP‐MS analysis. Complementation studies confirmed the role of CopX in USA300 copper hyper‐resistance in rich media. However, in more stressful conditions such as the restricted RPMI‐A medium, the pcopXL complementing plasmid inhibited growth in the presence of additional copper. This was also observed when the *copX* deletion mutant was complemented with a plasmid carrying *copX* only (Supporting Information Fig. S1). qRT‐PCR showed a large increase in expression of *copX* in the complementing strains and yet over‐expression of CopX is only inhibitory in the presence of high copper concentrations, suggesting that the cell cannot cope with large amounts of CopX in excess copper for reasons that are unclear.

Interestingly, unlike CopX, the similar *S. aureus* CopB P_1B‐3_ type transporter only confers copper‐hyper resistance when expressed at very high levels due to there being multiple copies on free‐replicating plasmids (Baker *et al*., 2011); unpublished data not shown). A single copy of *copXL* in *S. aureus* JE2 shows equivalent copper hyper‐resistance to *S. aureus* ATCC12600, which has *copB* on a multicopy plasmid. Our transcription data do not suggest that expression of *copXL* is notably higher than that of the single copy chromosomally encoded *copA* in wild type USA300. Therefore, together our data suggest that *S. aureus* CopX is likely a highly effective transporter, or there are other copper‐dependent factors involved.

The novel CopL lipoprotein is also important for copper hyper‐resistance, copper efflux and survival in macrophages. Homologues of this protein are found in many commensals and environmental bacteria and yet its function is unknown. It is predicted that CopL is surface bound and may bind copper via the tandem DUF1541 domains, though this remains to be tested. Thus, CopL could act as a surface‐bound copper sink that interacts with CopX or CopA. Alternatively CopL could act as a copper sensor and regulate copper‐dependent gene expression, thus increasing resistance to copper, analogous to the *E. coli* NlpE/CutF lipoprotein, which is proposed to induce expression of other genes involved in the adaptation to copper through interaction with the CpxA‐CpxR two‐component regulator system (Gupta *et al*., 1995).

Our data show that *S. aureus* strains that possess the *copXL* genes show copper hyper‐resistance compared to *S. aureus* strains that do not. Importantly CopX and CopL enhance intracellular survival in macrophages. This could explain why USA300 shows increased fitness in lower lung infection compared to hospital‐associated (HA)‐MRSA and improved survival in alveolar macrophages (Yajjala *et al*., 2016).

Importantly possession of the *copXL* locus is predicted to confer a strong selective advantage during infection, because copper is used as an antibacterial by the host and plays an essential role in innate immunity, virulence and colonization of the nasopharynx (Shafeeq *et al*., 2011; Chatterjee and Otto, 2013; Fu *et al*., 2014; Johnson *et al*., 2015; Hyre *et al*., 2017). Copper hyper‐resistance will also confer increased fitness clinically (Marcus *et al*., 2017) and in the community because copper toxicity is increasingly being exploited therapeutically as an antimicrobial. Therefore, acquisition of the *copXL* genes may in part explain the success of the *S. aureus* USA300 epidemic strain in both North and South America.

In conclusion, this study shows that acquisition of the *copXL* locus enhances *S. aureus* USA300 fitness due to increased resistance to innate immunity. This has worrying implications because *S. aureus* USA300 clones are spreading globally (Glaser *et al*., 2016; Dach *et al*., 2016; Planet *et al*., 2016), with emergences in Europe, Africa and India (Schaumburg *et al*., 2014; Egyir *et al*., 2015; Rajan *et al*., 2015). Consequently, our study shows the importance of the impact of acquisition of additional copper resistance mechanisms for pathogenic bacteria and the subsequent risks to our health and well‐being, and demonstrates the need for surveillance and efforts to combat copper resistance mechanisms.

## Experimental procedures

### Bacterial strains

All bacterial strains and *Staphylococcus aureus* mutants are listed in Supporting Information Table S1. *E. coli* was cultured in Luria broth (LB) and was handled and transformed using standard protocols. *S. aureus* strains were plated onto Luria agar (LA) and cultured statically at 37°C in 5% (v/v) CO_2_, in the chemically‐defined growth medium RPMI 1640 (Sigma‐Aldrich) supplemented with an amino acids solution (Sigma‐Aldrich) to a final concentration of 2% (v/v), termed RPMI‐A or Brain Heart Infusion (BHI) or Tryptic soy agar (TSA). Media were supplemented with tetracycline (5 µg ml^−1^), chloramphenicol (10 µg ml^−1^), ampicillin (100 µg ml^−1^), kanamycin (75 µg ml^−1^), spectinomycin (1250 µg ml^−1^) or erythromycin (10 µg ml^−1^) when required. For blue/white screening, 40 µl of 50 mg ml^−1^ X‐Gal was spread onto agar plates before inoculation with bacteria.

### Copper locus mutagenesis

The Nebraska Transposon Mutant Library (NTML) consists of 1920 defined mutants of non‐essential genes in *S. aureus* USA300 JE2 (Bose *et al*., 2013). JE2 transposon mutants NE590, NE191, NE561 and NE1750 (*copX, copL*, *copA* and *csoR* respectively) were transduced, using phage, into a clean USA300 JE2 background to eliminate any secondary mutations that may have been generated during library construction. The large transposon insertion in each mutant can be replaced with either an antibiotic selective marker or a markerless cassette using a set of molecular tools, as described by Bose and colleagues (2013). The transposon in *copX* was replaced with a markerless cassette, in *copL* with an *aphA‐3* kanamycin resistance gene, and in *copA* with an *aad9* spectinomycin resistance gene using the temperature sensitive vectors pTNT, pKAN and pSPC respectively (Bose *et al*., 2013), resulting in the production of new JE2 mutant strains designated *copX::TnT*, *copL::kanR* and *copA::specR*. The *copA::specR* mutation was also transduced into the *copX* mutant to create the double *copAX* mutant. The pcopXL complementation plasmid was constructed by cloning the complete *copXL* coding sequence alongside 551 bp upstream of *copX*, allowing transcription from the native promoter, into the *E. coli*/*S. aureus* shuttle vector pMK4 (Sullivan *et al*., 1984). Briefly, the *copXL* sequence was PCR amplified from USA300 JE2 wildtype DNA using primers copX_F2 and copL_R, which contained EcoRI and BamHI restriction sites respectively. The resulting PCR product was digested and ligated into pMK4 at these sites, transformed into *S. aureus* strain RN4220 and subsequently phage transduced into each recipient strain.

For construction of a clean deletion mutant of *copX*, the temperature sensitive plasmid pIMAY that utilizes a *secY* antisense counter‐selection determinant for allelic replacement was used (Monk *et al*., 2012; Monk and Foster, 2012). Briefly, flanking regions of *copX* were amplified by PCR using primer pairs OL961F/962R and OL963F/964R with *S. aureus* FPR3757 genomic DNA as the template and PrimeStar Max DNA polymerase (Takara). A ‘crossover’ PCR was done with the primers OL961F and OL964R using the upstream and downstream PCR products as templates. After subcloning into pCR2.1 (Invitrogen), the fragment was cloned into pIMAY using NotI and EcoRI restriction sites and the recombinant plasmid transformed into chemically competent *E. coli* SA08B (Lucigen) (Monk *et al*., 2012). The plasmid was isolated from *E. coli*, verified by sequencing and then electroporated into *S. aureus* FPR3757, which was plated onto TSA plus Cm 10 μg ml^−1^ and incubated at 28°C. Transformants were incubated on TSA plus Cm 10 μg ml^−1^ at 37°C to stimulate pIMAY chromosomal integration. Colonies free of replicating plasmid were grown at 28°C without Cm and then plated onto TSA containing anhydrotetracycline 1 μg ml^−1^ for counter‐selection. Resulting colonies that showed loss of Cm resistance were screened by colony PCR for the absence of *copX*. Putative mutants were validated by PCR amplification of genomic DNA flanking the deletion and DNA sequencing (Monk *et al*., 2012).

The pcopX complementation plasmid was constructed by cloning a 2.2 kb fragment PCR product using primers 944F and 947R, that contained *copX* and its predicted promoter (–196 bp upstream from start codon) into the *E. coli/S. aureus* shuttle vector pOS1, provided by Dr. V.J. Torres at New York University School of Medicine. The amplified fragment was cloned into the EcoRI and BamHI restriction sites of pOS and then transformed into *E. coli* SA08B (Lucigen). The plasmid was isolated from *E. coli*, verified by sequencing and electroporated into the S. aureus FRP3757 *copX* deletion strain. Transformants were selected with Cm 10 μg ml^−1^. The presence of *copX* was confirmed by colony PCR and subsequent plasmid extraction and digestion with BamHI and EcoRI.

### Copper growth assay

Strains were incubated in 20 ml RPMI‐A or BHI for 16 h at 37°C and 5% (v/v) CO_2_. Cells were pelleted and re‐suspended in 1 ml of fresh RPMI‐A or BHI and used to inoculate fresh, prewarmed RPMI‐A or BHI to an OD_600nm_ of 0.05. Aliquots (10 ml) were taken and supplemented CuCl_2_ and grown for 24 h. Subinhibitory and toxic copper concentrations were determined using OD_600_ as a measure of growth after 24 h. For copper toxicity growth assays, RPMI‐A was supplemented with 500 μM CuCl_2,_ while BHI was supplemented with 2 mM CuCl_2_. Control conditions without additional copper were included in all experiments. Supplementation with 100 μM was determined to be subinhibitory in RPMI‐A and used for subsequent experiments were subinhibitory conditions were required.

### Whole cell metal analysis by ICP‐MS

Strains were incubated in RPMI‐A for 16 h and then inoculated into RPMI‐A to an OD_600nm_ of 0.05. Aliquots were made from these subcultures and supplemented with 100 µM CuCl_2_, Fe_2_(SO_4_)_3_ or ZnCl_2_ with a fourth aliquot prepared with no metal supplementation. Samples were normalized to the same OD_600nm_, harvested by centrifugation, and washed twice in 50mM Tris, pH 7.5, 100 mM NaCl, 10 mM EDTA at 4°C. Washed pellets were stored at −80°C until use, then thawed and digested with concentrated nitric acid (Merck) for 48 h. Digests were centrifuged at 21 000 *g* for 20 min at 4°C and the supernatants were analysed for total metal content using inductively coupled plasma mass spectrometry (ICP‐MS; Thermo x‐series). Samples were diluted 10‐fold in 2% nitric acid containing 20 μg l^−1^ platinum and silver as internal standards, and analysed (100 reads, 30 ms dwell, 3–5 channels, 0.02 atomic mass unit separation, in triplicate) for ^56^Fe, ^65^Cu, ^66^Zn, ^107^Ag and ^195^Pt in collision cell mode (3 ml min^−1^ 8% H_2_ in He collision gas), and metal concentrations determined by comparison to matrix‐matched elemental standard solutions.

### RNA extraction

Strains were grown to mid‐exponential phase in RPMI‐A, with and without 100 µM CuCl_2_. Bacteria were pelleted and re‐suspended in 1 ml PBS, and 200 µl of 19:1 ethanol:phenol was added to preserve the RNA during storage. Bacteria were pelleted and lysed for 30 min at 37°C in 200 µl TE buffer containing 10 µg ml^−1^ lysostaphin and 20 µg ml^−1^ proteinase K, before being homogenized in RLT buffer (Qiagen) containing 10 µl ml^−1^ β‐mercaptoethanol, using a MP Biomedical Fast Prep Instrument and Lysing Matrix B tubes (MP Biomedicals). RNA was purified from this lysate using a Qiagen RNeasy kit, with both on column DNase treatment (Qiagen) and a subsequent TurboDNase step (Thermo Fisher) to ensure RNA purity. All RNA samples were confirmed to be DNA‐free using qPCR.

### qRT‐PCR

Total RNA (1 µg) was converted into cDNA using Superscript IV VILO Master Mix reverse transcriptase (Invitrogen), and from this 0.5 ng of cDNA was used per qPCR reaction. qPCR was carried out using SYBR Green Master mix (Applied Biosystems) in a 7300 Fast System (Applied Biosystems) following manufacturer's instructions. Relative gene expression for each of the sample genes (for primers and concentrations used see Supporting Information Table S2) was normalized to the expression of the endogenous control gene *gyrB*, and calculated in relation to expression of a calibrator strain, JE2, without copper, using the ΔΔCt method to calculate RQ (2^‐ΔΔCt^) (Livak and Schmittgen, 2001). Results are the product of 3 independent biological replicates, and samples were run in technical triplicate within each experiment. Statistical significance was calculated using 2‐way ANOVA with Dunnett's multiple comparison test.

### Cloning, expression and purification of recombinant CsoR

The wild type *csoR* gene was amplified from *S. aureus* genomic DNA by PCR using primers CsoR_F and CsoR_R (Supporting Information Table S2) and the PCR product cloned into pGEM‐T (Promega). An internal *Nde*I site was removed through silent mutation (CAT to CAC in the His76 codon) using primers CsoR_NdeI‐F and CsoR_NdeI‐R (Supporting Information Table S2), before subcloning of the insert into *Nde*I/*Bam*HI digested pET‐29a (Novagen) to generate pET29a‐CsoR, confirmed by sequencing (GATC). *E. coli* BL21 (λDE3) cells transformed with pET29a‐CsoR were cultured in LB at 37°C and protein expression induced at OD_600_ ∼0.6 for 5 h at 30°C with 1 mM isopropyl‐β‐D‐L‐thiogalactopyranoside (IPTG). Cells were harvested by centrifugation (4000 *g*, 30 min, 4°C), washed in 25 mM Tris, 10 mM EDTA, pH 7.5 and lysed by sonication in 25 mM Tris, 15 mM dithiothreitol (DTT), pH 8.5 containing protease inhibitor cocktail (Sigma). The lysate was clarified by centrifugation (48 000 *g*, 40 min, 4°C), the supernatant filtered (0.45 µm) and diluted sixfold in the same buffer, before purification on a 5 ml HiTrap Q HP anion exchange (AEX) column (Ge Healthcare) using an Akta fast performance liquid chromatography (FPLC) system (GE Healthcare). Protein was eluted with a linear NaCl gradient (0–1 M) over 10 column volumes (CV) and fractions analysed by SDS‐PAGE. Fractions identified by SDS‐PAGE to contain CsoR were pooled, diluted to 50 mM NaCl, and concentrated by heparin affinity chromatography (GE Healthcare) eluted with 10 mM phosphate buffer, pH 7, 15 mM DTT, 1 M NaCl. Protein was treated with 10 mM EDTA and 20 mM Tris(2‐carboxyethyl)phosphine (TCEP) at 4°C overnight, and resolved on a Superdex 75 16/600 gel filtration column (GE Healthcare) in 25 mM Hepes, 200 mM NaCl, 15 mM DTT, pH 7.5. Resulting CsoR fractions were concentrated using 15 ml a 3MWCO centrifugal filter device (Amicon), flash‐frozen in liquid nitrogen and stored at −80°C.

### EMSA

DNA regions (∼200 bp) upstream of *copX*, *copL* and *copA* were amplified from *S. aureus* genomic DNA by PCR using the relevant primer pairs (see Supporting Information Table S2), cloned into pGEM‐T, and confirmed by sequencing. These (and empty pGEM‐T for the control DNA) were used in PCRs using M13 primers to produce DNA samples for each operator/promoter region, which were purified (PCR clean‐up kit; Sigma). DTT was removed from recombinant CsoR in an anaerobic ([O_2_] < 5 ppm) chamber (Belle Technology) by binding to a 1 ml AEX column and washing with 10 CV anaerobic 20 mM Hepes, pH 7.5, then eluting in this buffer with 1 M NaCl. CsoR was quantified from absorbance at 280 nm using an experimentally‐determined (by amino acid analysis – Alta Biosciences) extinction coefficient, (*ɛ*_280nm_=1490 M^−1^ cm^−1^), and the 5,5`‐dithio‐bis‐(2‐nitrobenzoic acid) (DTNB) (Sigma) assay for free thiols (Vita *et al*., 2015). Stock solutions (100 mM) of Cu(I) were prepared as previously described (Vita *et al*., 2015), and 1 mole equivalent was added to CsoR anaerobically and incubated for 30 min at room temperature. EMSA reactions contained 100 ng µl^−1^ poly(dI‐dC) (Sigma), 1 mM DTT, 0.4 mg ml^−1^ bovine serum albumin (BSA), 100 nM target DNA and 100 nM control DNA in 20 mM Hepes, 100 mM NaCl, pH 7.0. Apo‐ or Cu(I)‐CsoR was added (0–100 µM), and the mixture equilibrated anaerobically for 30 minutes at room temperature, before resolution on 6% (w/v) native polyacrylamide gels (pre‐electrophoresed for 15 min at 42 V) for 60–80 min at 82 V. Gels were stained in 10% (v/v) SYBR Safe (Invitrogen).

### Macrophage survival assays

The RAW264.7 mouse macrophage cell‐line was cultured in DMEM 10% (v/v) FBS. Monolayers were prepared by seeding 2 × 10^6^ cells per ml (500 µl per well) in 24‐well plates, which were then cultured for 24 h in serum‐free DMEM supplemented with copper sulfate (20–40 µM) and mouse IFNɣ (50 µg ml^−1^, Gibco) for 18 h at 37°C, 5% CO_2_. Immediately before infection, RAW264.7 cells were washed with DMEM alone. *S. aureus* strains were cultured statically in RPMI‐1640 supplemented with 100 µM copper sulfate for 18 h at 37°C, 5% CO_2_. Immediately before the experiment bacteria were washed twice with DMEM and adjusted to an OD_600_ ∼ 0.05 (ca. 2 × 10^7^ CFU ml^−1^) in DMEM and co‐incubated with monolayers for 30 min. The monolayers were washed and extracellular bacteria killed by treatment with gentamicin (200 µg ml^−1^) & lysostaphin (100 µg ml^−1^) for 30 min. Monolayers were washed with DMEM and then lysed with ice‐cold water at time point 0 (T0) and after additional 3 h incubation (T3) to determine the survival rates (CFU ml^−1^), lysates plated on agar, and CFUs counted.

### Statistical analysis

Data are summarized as standard error of the mean (SEM.) and were analysed using GraphPad Prism version 6.04 (GraphPad Software, La Jolla, CA). ANOVA or *t* tests were used to determine significance as appropriate.

## Supporting information

Additional Supporting Information may be found in the online version of this article at the publisher's web‐site:

**Table S1.** Bacterial strains and plasmids used in this study.

**Table S2.** Primers used in this study.

**Fig. S1.** Growth of wild‐type *S. aureus* JE2 and FRP with that of the isogenic copper mutants and their complementation strains in both RPMI‐A and BHI ± toxic concentration of copper. In each case, optical densities at 600 nm were determined after 24 h of growth at 37°C for 24 h in 5% (v/v) CO2. Error bars represent ±1 SEM of 3 independent biological repeats. Significance of JE2 growth compared to other strains was determined with a two‐way ANOVA. Dashed lines on graphs represent the starting optical density. There is no significant difference in growth of these strains in the absence of copper in either medium.
**Fig. S2.** (A) A schematic representing the position and sequence of the qRT‐PCR amplicons used in the qRTPCR analysis, in relation to the *copXL* operon. (B) Transcription of *copA, copX, copL* and the intergenic region between *copX* and *copL* (copXL) was determined by qRT‐PCR in cells cultured in the presence or absence of subinhibitory CuCl2 (100 μM) during exponential growth in JE2, *copA::spec*, *copX::TnT* and JE2 *csoR:*:FNS. In addition, *copX* expression was also measured in the USA300 strain FPR3757 and *copL* expression in FRP3575 and isogenic *copX* deletion mutant to show that *copL* transcription is unaffected in this strain. Data are presented on a log10 scale to highlight decreased expression of *copL* in the *copX* mutant. (C) Transcription of *copX* in JE2 and the *copX* insertion mutant carrying the complementation construct pcopXL. In each case, relative expression was calculated as RQ using the ΔΔCt method, which normalizes expression in each strain against an endogenous control gene (*gyrB*) and expresses the data relative to a reference strain (JE2 in the absence of Cu). Error bars represent ± 1 SEM of 3 biological repeats (each performed in technical triplicate). The dashed line represents the RQ of 1, which represents the baseline level of expression for the calibrator strain JE2 grown without copper. Data are presented on a log10 scale, and the dashed line at *y* = 1 indicates the expression level in the reference strain. Significance of expression in each strain compared to the reference was determined by two‐way ANOVA with Dunnett's multiple comparison test * = *p* ≤ 0.0001.
**Fig. S3.** Macrophage intracellular bacterial survival CFU data. Mouse RAW264.7 macrophages were seeded at 2 × 10^6^ per ml in DMEM into 24‐well plates and activated with mouse IFN‐γ and Cu2SO4 for 18 h. Bacteria were added at a MOI of 10 in DMEM and co‐incubated with monolayers for 30 min. The monolayers were subsequently washed and extracellular bacteria were killed by treatment with gentamycin (200 μg ml^−1^) and lysostaphin (100 μg ml^−1^) for 30 min. To quantify intracellular bacteria, monolayers were washed and lysed with ice‐cold water at time point 0 (T0) and after 3 h (T3). CFUs were counted to determine numbers of viable bacteria. Bars represent the mean CFU/ml ± SD for three independent experiments.
