# Supplementary material for: A horizontally gene transferred copper resistance locus confers hyper‐resistance to antibacterial copper toxicity and enables survival of community acquired methicillin resistant Staphylococcus aureus USA300 in macrophages
Source: Environ Microbiol. 2018 Mar 26;20(4):1576–89. doi: 10.1111/1462-2920.14088 (PMC5947656; doi:10.1111/1462-2920.14088)
Supplement: Supplementary file 1 — Table S1. Bacterial strains and plasmids used in this study. [file EMI-20-1576-s001.doc]

**Table S 1.** Bacterial strains and plasmids used in this study.

| **Strain/plasmid** | **Description** | **Source** |
| --- | --- | --- |
| *S. aureus* |  |  |
| JE2 | USA300 CA-MRSA strain LAC without plasmids | (Fey et al. 2013) |
| RN4220 | Highly transformable, restriction deficient *S. aureus* |  |
| NE590 | JE2 *copX::ΦNΣ* | (Bose et al. 2013) |
| NE1913 | JE2 *copL::ΦNΣ* | (Bose et al. 2013) |
| NE561 | JE2 *copA::ΦNΣ* | (Bose et al. 2013) |
| copX | JE2 *copX::pTNT* | This study |
| copA | JE2 *copA::aad9 (specR)* | This study |
| copL | JE2 *copL::aphA-3 (kanR)* | This study |
| csoR | JE2 *csoR::ΦNΣ* | (Bose et al. 2013) |
| FPR3757 | USA300 CA-MRSA strain LAC with plasmids | (Fey et al. 2013) |
| copX deletion | FPR3757 *copXΔ* | This study |
| Newman | Wild type | (Duthie & Lorenz 1952) |
| SH1000 | 8325-4 with *rsbU* mutation repair | (Horsburgh et al. 2002) |
| EMRSA-16 | PM64, clinical isolate | (Moore & Lindsay 2002) |
| ATCC12600 | Wild type | (Sitthisak et al. 2005) |
| DP | MRSA 078588D | (Ward et al. 2014) |
| JH | MRSA 072736J | (Ward et al. 2014) |
| *E. coli* |  |  |
| DH5α | Strain used for cloning experiments |  |
| BL21 | Strain used for protein expression | Novagen |
| *Plasmids* |  |  |
| pTNT | pJB38 with homologous DNA to *bursa aurealis* | (Bose et al. 2013) |
| pSPC | pTNT with *aad9* | (Bose et al. 2013) |
| pKAN | pTNT with *apaH-3* | (Bose et al. 2013) |
| pMK4 | Low copy shuttle vector used for complementation | (Sullivan et al. 1984) |
| pcopXL | pMK4 with *S. aureus copXL* | This study |
| pcopX | pOS with *S. aureus copX* | This study |

**Supplementary References**

Bose, J.L., Fey, P.D. & Bayles, K.W., 2013. Genetic tools to enhance the study of gene function and regulation in *Staphylococcus aureus.* *Applied and Environmental Microbiology*, 79(7), pp.2218–2224.

Duthie, E.S. & Lorenz, L.L., 1952. Staphylococcal coagulase; mode of action and antigenicity. *Journal of General Microbiology*, 6(1-2), pp.95–107.

Fey, P.D. et al., 2013. A genetic resource for rapid and comprehensive phenotype screening of nonessential Staphylococcus aureus genes. *mBio*, 4(1), pp.e00537–12.

Horsburgh, M.J. et al., 2002. sigmaB Modulates Virulence Determinant Expression and Stress Resistance: Characterization of a Functional *rsbU* Strain Derived from *Staphylococcus aureus* 8325-4. *Journal of bacteriology*, 184(19), pp.5457–5467.

Moore, P.L.C. & Lindsay, J.A., 2002. Molecular characterisation of the dominant UK methicillin-resistant *Staphylococcus aureus* strains, EMRSA-15 and EMRSA-16. *Journal of Medical Microbiology*, 51(6), pp.516–521.

Sitthisak, S. et al., 2005. Characterization of a multicopper oxidase gene from *Staphylococcus aureus.* *Applied and Environmental Microbiology*, 71(9), pp.5650–5653.

Sullivan, M.A., Yasbin, R.E. & Young, F.E., 1984. New shuttle vectors for Bacillus subtilis and Escherichia coli which allow rapid detection of inserted fragments. *Gene*, 29(1-2), pp.21–26.

Ward, M.J. et al., 2014. Time-Scaled Evolutionary Analysis of the Transmission and Antibiotic Resistance Dynamics of *Staphylococcus aureus* Clonal Complex 398. *Applied and Environmental Microbiology*, 80(23), pp.7275–7282.
