# Supplementary material for: A horizontally gene transferred copper resistance locus confers hyper‐resistance to antibacterial copper toxicity and enables survival of community acquired methicillin resistant Staphylococcus aureus USA300 in macrophages
Source: Environ Microbiol. 2018 Mar 26;20(4):1576–89. doi: 10.1111/1462-2920.14088 (PMC5947656; doi:10.1111/1462-2920.14088)
Supplement: Supplementary file 2 — Table S2. Primers used in this study. [file EMI-20-1576-s002.doc]

**Table S2. Primers used in this study**.

| **Name** | **Nucleotide sequence (5’ to 3’)^a^** | | **Restriction site** |
| --- | --- | --- | --- |
| CsoR_F | GGGCATATGACTGAACAAGATAATGC | |  |
| CsoR_R | GGGGGATCCTTAGTCTTTAATCAATTTTTGAA | |  |
| CsoR_NdeI_F | GATAAAGTTATTAGAACAACACATGAAAAGTTGTATTATG AATAAAG | | Nde I |
| CsoR_NdeIR | CTTTATTCATAATACAACTTTTCATGTGTTGTTCTAATAACTTTATC | | Nde I |
| **EMSA** |  | |  |
| PcopA_F | GGGGGATCCATTTCTTTTAAGTCACCTAAG | |  |
| PcopA_R | GGGGATCCCACATTATTGCCTCCCTGT |  | |
| PcopX_F | GGGGGATCC ctt taa gaa tat taa tat atg |  | |
| PcopX_R | GGGGGATCC CAA att aac ccc tcc taa tg |  | |
| PcopL_F | GGGGGATCC agt aat cca tca gat atc att c |  | |
| PcopL_R | GGGGGATCC CAT aat aaa act cct atc |  | |
| **Mutagenesis** |  |  | |
| copA MF | GAATAAACTTGATGACGTTAATG |  | |
| copA MR | TCGGCTCTAGCTTCTAAATAC |  | |
| copX MF | GGTGAAGAGCATAATCATC |  | |
| copX MR | CGACGATATCATCAGTCATG |  | |
| copL MF | GGGTGCAGGTTATAATATTG |  | |
| copL MR | AGTATCGCCCTTACTAAATC |  | |
| **qRT-PCR** |  |  | |
| gyrB-F | GACTGATGCCGATGTGGA |  | |
| gyrB-R | AACGGTGGCTGTGCAATA |  | |
| copA_qRT_F | TAAATAAAATGGACGGCGTTC |  | |
| copA_qRT_R | AATGCGAGTGACAAGTTTATCAG |  | |
| copX_qRT_F | GCACAACTCATGCCAGAAGA |  | |
| copX_qRT_R | TTGATACCGTCTCCGACCAT |  | |
| copL_qRT_F | GCCAGGTATGAAAGGTGCAG |  | |
| copL_qRT_R | TCTCTTCGTTTACGACCCATTT |  | |
| copXL_qRTF | TCGTTGCAATTAATGCCTTT |  | |
| copXL_qRTR | CATCATTATTGGAGCAAGCTGA |  | |
| copZ_qRT_F | GTTGAAGGTATGAGCTGTGGTC |  | |
| copZ_qRT_R | TCAGCTGAAGTGACACCGTC |  | |
| **Complementation** | |  | |
| copX_F2 | CT**GAATTC**GAGGTTGTATATAACTCAC | EcoRI | |
| copL_R | TT**GGATCC**CAATTTAGAACTAGATTTTTATC | BamHI | |
| OL961F | CCGGAATTCGTGACATCACGTGCTATTTCAT |  | |
| 962R | ATCATAATAAAACTCCTATCGTGATTTTGATGATTATGCTCTTC |  | |
| OL963F | GATAGGAGTTTTATTATGATTAAAAAATTATTTTTTATG |  | |
| 964R | AAGGAAAAAAGCGGCCGCCGCCTCTGCGTATCGGTTA |  | |
| 944F | TTAAATATCGATGACAAGGTC |  | |
| 947R | CGGGATCCCTCCTATCTTTTATTTTAATTTTAATG |  | |
