# Supplementary material for: A horizontally gene transferred copper resistance locus confers hyper‐resistance to antibacterial copper toxicity and enables survival of community acquired methicillin resistant Staphylococcus aureus USA300 in macrophages
Source: Environ Microbiol. 2018 Mar 26;20(4):1576–89. doi: 10.1111/1462-2920.14088 (PMC5947656; doi:10.1111/1462-2920.14088)
Supplement: Supplementary file 3 — Fig. S1. Growth of wild‐type S. aureus JE2 and FRP with that of the isogenic copper mutants and their complementation strains in both RPMI‐A and BHI ± toxic concentration of copper. In each case, optical densities at 600 nm were determined after 24 h of growth at 37°C for 24 h in 5% (v/v) CO2. Error bars represent ±1 SEM of 3 independent biological repeats. Significance of JE2 growth compared to other strains was determined with a two‐way ANOVA. Dashed lines on graphs represent the starting optical density. There is no significant difference in growth of these strains in the absence of copper in either medium. Fig. S2. (A) A schematic representing the position and sequence of the qRT‐PCR amplicons used in the qRTPCR analysis, in relation to the copXL operon. (B) Transcription of copA, copX, copL and the intergenic region between copX and copL (copXL) was determined by qRT‐PCR in cells cultured in the presence or absence of subinhibitory CuCl2 (100 μM) during exponential growth in JE2, copA::spec, copX::TnT and JE2 csoR::FNS. In addition, copX expression was also measured in the USA300 strain FPR3757 and copL expression in FRP3575 and isogenic copX deletion mutant to show that copL transcription is unaffected in this strain. Data are presented on a log10 scale to highlight decreased expression of copL in the copX mutant. (C) Transcription of copX in JE2 and the copX insertion mutant carrying the complementation construct pcopXL. In each case, relative expression was calculated as RQ using the ΔΔCt method, which normalizes expression in each strain against an endogenous control gene (gyrB) and expresses the data relative to a reference strain (JE2 in the absence of Cu). Error bars represent ± 1 SEM of 3 biological repeats (each performed in technical triplicate). The dashed line represents the RQ of 1, which represents the baseline level of expression for the calibrator strain JE2 grown without copper. Data are presented on a log10 scale, and the dashed line at y = 1 [file EMI-20-1576-s003.pdf]

S1

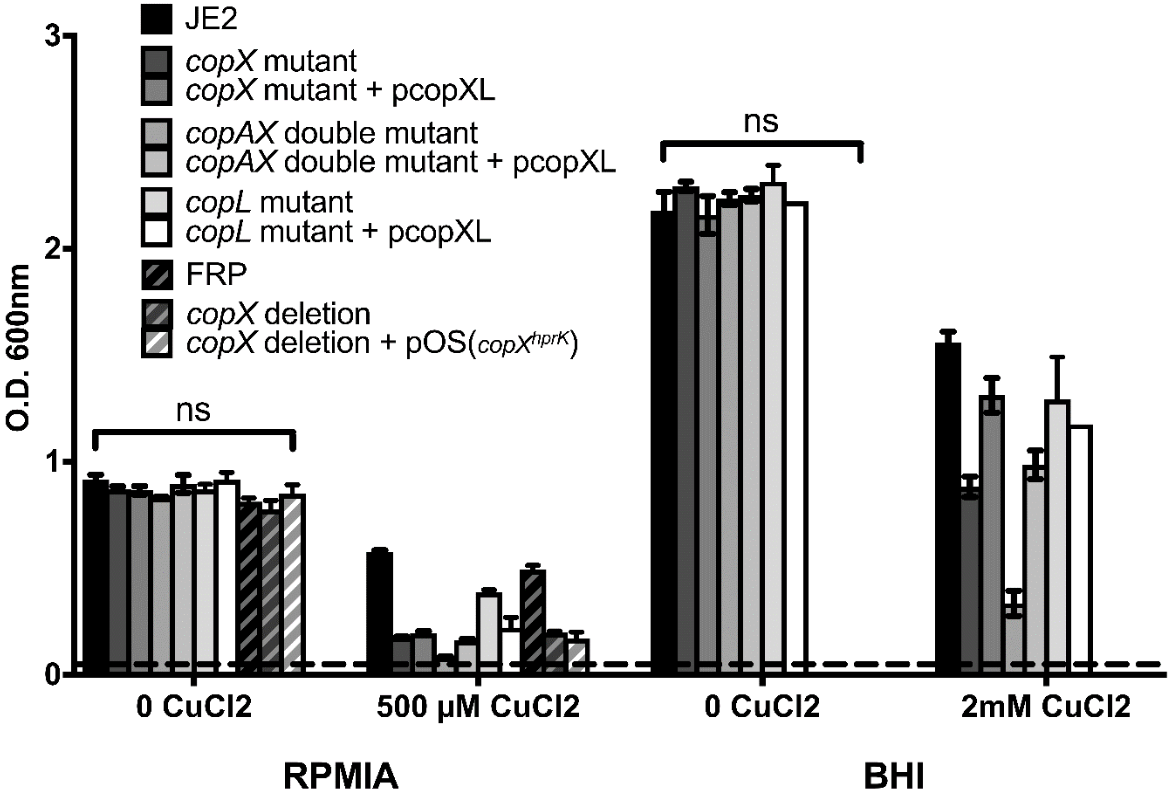

Supplementary Figure 1

Growth of wild-type *S. aureus* JE2 and FRP with that of the isogenic copper mutants and their complementation strains in both RPMI-A and BHI +/- toxic concentration of copper. In each case, optical densities at 600 nm were determined after 24h of growth at 37°C for 24 hours in 5 % (v/v) CO_2. Error bars represent ± 1 S.E.M. of 3 independent biological repeats. Significance of JE2 growth compared to other strains was determined with a two-way ANOVA. Dashed lines on graphs represent the starting optical density. There is no significant difference in growth of these strains in the absence of copper in either medium.

S2

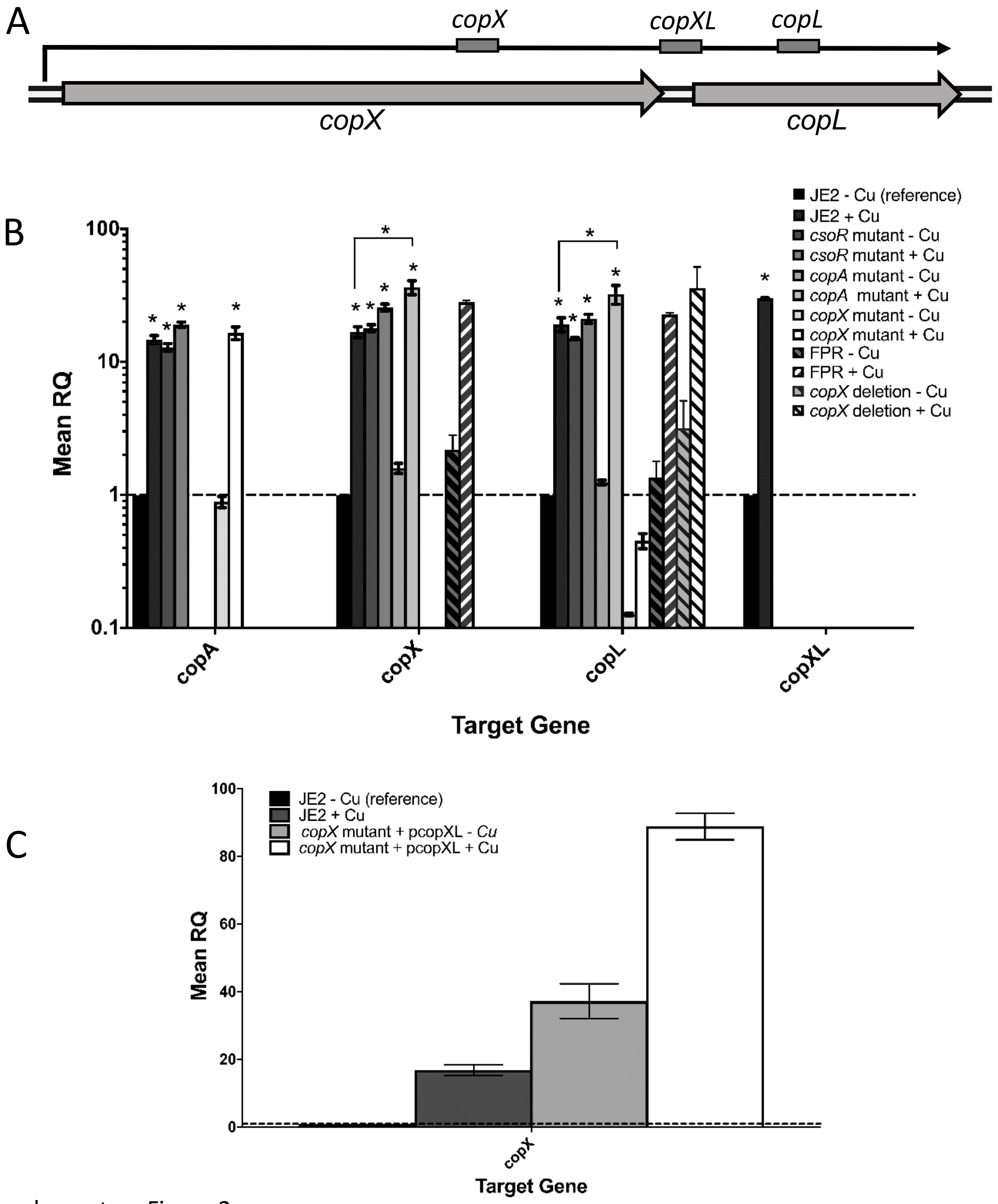

Supplementary Figure 2.
(A) A schematic representing the position and sequence of the qRT-PCR amplicons used in the qRT-PCR analysis, in relation to the *copXL* operon. (B) Transcription of *copA, copX, copL* and the intergenic region between *copX* and *copL* (copXL) was determined by qRT-PCR in cells cultured in the presence or absence of sub-inhibitory $CuCl_2$ (100 µM) during exponential growth in JE2, *copA::spec*, *copX::TnT* and JE2 *csoR::ΦΝΣ*. In addition, *copX* expression was also measured in the USA300 strain FPR3757 and *copL* expression in FRP3575 and isogenic *copX* deletion mutant to show that *copL* transcription is unaffected in this strain. Data is presented on a log10 scale to highlight decreased expression of *copL* in the *copX* mutant. (C) Transcription of *copX* in JE2 and the *copX* insertion mutant carrying the complementation construct pcopXL. In each case, relative expression was calculated as RQ using the ΔΔCt method, which normalises expression in each strain against an endogenous control gene (*gyrB*) and expresses the data relative to a reference strain (JE2 in the absence of Cu). Error bars represent ± 1 S.E.M. of 3 biological repeats (each performed in technical triplicate). The dashed line represents the RQ of 1, which represents the baseline level of expression for the calibrator strain JE2 grown without copper. Data is presented on a Log10 scale, and the dashed line at y=1 indicates the expression level in the reference strain. Significance of expression in each strain compared to the reference was determined by 2-way ANOVA with Dunnett's multiple comparison test * = p≤0.0001.

S3

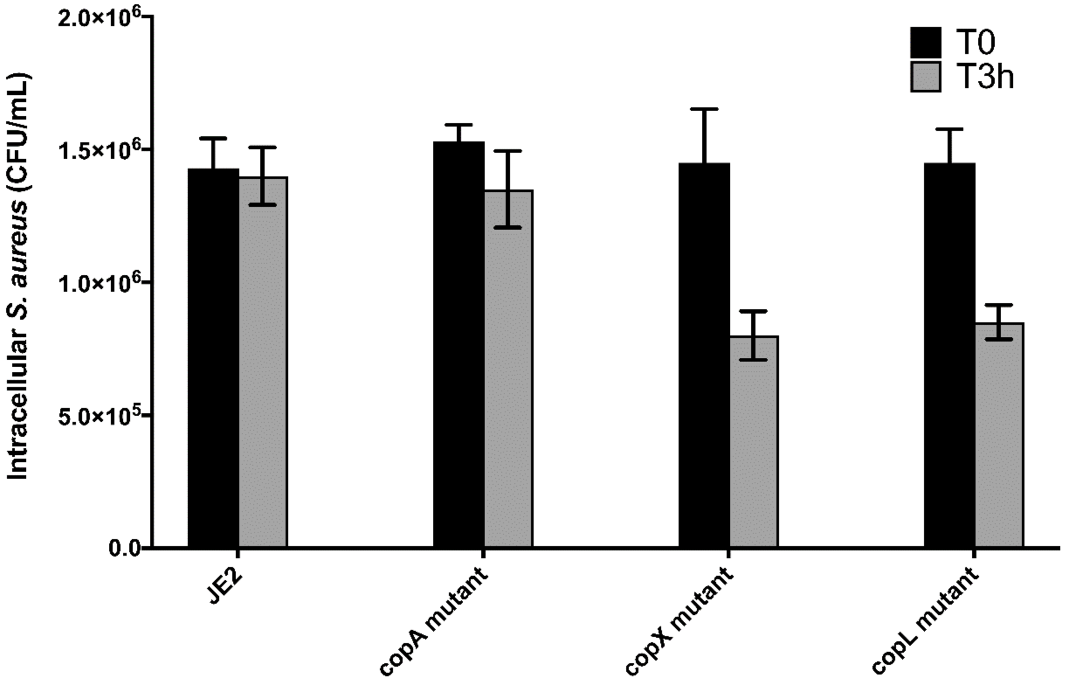

Supplementary Figure 3

Macrophage intracellular bacterial survival CFU data. Mouse RAW264.7 macrophages were seeded at $2×10^6$ per ml in DMEM into 24-well plates and activated with mouse IFN-γ and Cu_2SO_4 for 18h. Bacteria were added at a multiplicity of infection (MOI) of 10 in DMEM and co-incubated with monolayers for 30 min. The monolayers were subsequently washed and extracellular bacteria were killed by treatment with gentamycin (200 µg mL^{-1}) and lysostaphin (100 µg mL^{-1}) for 30 min. To quantify intracellular bacteria, monolayers were washed and lysed with ice-cold water at time point 0 (T0) and after 3 h (T3). CFUs were counted to determine numbers of viable bacteria. Bars represent the mean CFU/ml ± SD for three independent experiments.
